# Brain and physiological responses to flavored waters with different sweeteners: a randomized crossover study in healthy young adults

**DOI:** 10.1016/j.ajcnut.2026.101401

**Published:** 2026-06-20

**Authors:** Paul AM Smeets, Ralf Veit, Els Oosterink, Saskia Meijboom, Davide Risso, Hubert Preissl, Stephanie Kullmann

**Affiliations:** 1 Division of Human Nutrition and Health, Wageningen University & Research, Wageningen, The Netherlands; 2 Institute for Diabetes Research and Metabolic Diseases (IDM) of the Helmholtz Center Munich at the University of Tübingen, Tübingen, Germany; 3 German Center for Diabetes Research (DZD), Tübingen, Germany; 4 Wageningen Food and Biobased Research, Wageningen University & Research, Wageningen, Netherlands; 5 Tate & Lyle PLC, London, UK; 6 Internal Medicine IV, Department of Diabetology, Endocrinology and Nephrology, University Hospital Tübingen, Tübingen, Germany; 7 Institute of Pharmaceutical Sciences, Department of Pharmacy and Biochemistry, Interfaculty Centre for Pharmacogenomics and Pharma Research at the Eberhard Karls University Tübingen, Tübingen, Germany

**Keywords:** sweeteners, brain imaging, cerebral blood flow, gastric emptying, glucose, insulin, appetite, magnetic resonance imaging

## Abstract

**Background:**

Drinks with low-no-calorie sweeteners (LNCSs) do not contribute to energy intake but still provide a hedonic experience through sweetness. LNCS can have differential effects on brain areas involved in food intake and reward compared with sugars.

**Objectives:**

This study determined changes in brain activity and the effect on physiological markers following the ingestion of flavored waters sweetened with the sugar sucrose or LNCS.

**Methods:**

Thirty healthy individuals participated in a randomized crossover study with 6 treatments. Participants were scanned after an overnight fast using magnetic resonance imaging, including arterial spin labeling to measure cerebral blood flow (CBF), before and after ingestion of 500-mL drinks: water, or equisweet flavored waters with 25 g sucrose, sucralose, stevia extract, allulose+stevia extract, or monk fruit extract. CBF was measured at baseline and 5 and 30 min; gastric content volume was measured at baseline and 25 and 45 min. Serum insulin and glucose were measured, and participants rated their appetite and thirst throughout each visit. Data were analyzed with linear mixed models.

**Results:**

Primary: Hypothalamus CBF was not differentially affected by any of the drinks. In the ventral tegmental area (midbrain), treatment effects differed, with lower ΔCBF after sucrose than water, sucralose and monk fruit drink ingestion at 30 min (difference: ∼8% ± 3%; all *P*_FDR_ < 0.05). Exploratory whole-brain analyses showed increased CBF after consumption for allulose+stevia (amygdala) and stevia (putamen) compared with sucrose (*P*_FWE_ < 0.05). Despite its low energy content, allulose+stevia delayed gastric emptying similar to sucrose, whereas only sucrose increased glucose and insulin concentrations.

**Conclusions:**

Although flavored waters with LNCS mostly elicit similar neural and gastrointestinal responses as water, they have some distinct effects on the brain compared with 25 g of sucrose, particularly in reward-related brain areas. Further exploration of the neural and physiological effects of allulose and stevia and dose-dependent investigations are warranted.

This study was registered at clinicaltrials.gov as NCT05575687 (https://clinicaltrials.gov/study/NCT05575687).

## Introduction

Because of the global obesity burden, there is great interest in strategies to reduce energy intake [1]. One common strategy is to replace sugar with low-no-calorie sweeteners (LNCSs). This strategy is prominently applied in soft drinks because sugar-sweetened soft drinks contribute to overconsumption and obesity [2,3]. There has been much controversy about potentially undesirable effects of providing sweetness, with or without calories, but empirical evidence does not support that exposure to sweetness stimulates a greater liking and desire for sweetness and greater intake of sweet foods [[4], [5], [6], [7]]. A wealth of evidence in humans suggests that overall nonnutritive-sweetened drinks are equivalent to water, in terms of their acute metabolic effects and their effects on energy intake and weight [6,7]. However, there is mounting evidence that not all LNCS have the same physiological effects because they are processed differently in the body [[8], [9], [10]]. This is also the case for various types of sugars that can elicit different glycemic responses [11]. Differences among nonnutritive sweeteners start with differences in their sensory properties [12,13] and are also apparent in their metabolic pathways and effects on the gut microbiota [8,14]. Interestingly, it has been shown that blocking glucose taste sensation decreases the speed of gastric emptying after ingestion of glucose [15] but not that of the nonnutritive sweetener aspartame [16].

The brain plays a crucial role in regulating energy homeostasis and eating behavior [17]. Several brain imaging studies have sought to examine the brain responses to simple solutions or drinks with sugars, LNCS, or only calories (nonsweet carbohydrates; maltodextrin) [[18], [19], [20], [21]]. Although some of these studies point to a difference between sugar and LNCS, the results are not very consistent. One potential limitation of such taste functional magnetic resonance imaging (fMRI) studies is the repeated administration of small sips over an extended period, which does not reflect naturalistic consumption patterns. Examining the response to ingestion of an amount that is regularly consumed (200–500 mL) is more ecologically relevant. Most of the studies using this approach have used blood oxygen level–dependent (BOLD) fMRI, the same magnetic resonance imaging (MRI) measure that is used in taste cue fMRI studies. Their most consistent finding is a decrease in the BOLD signal in the hypothalamus after ingestion of 75 g of sugar but not water [[22], [23], [24], [25], [26], [27]]. This decrease has been considered a biomarker for satiety, reflecting the reduced drive to eat after nutrient intake [28]. Conversely, an increase in hypothalamic activity in response to fructose has been observed in some studies and is thought to reflect incomplete appetite suppression [28]. However, with respect to different LNCS, there are few studies and these do not provide consensus with regard to consumption-induced brain activity changes [26,29]. Meyer-Gerspach et al. [30] used arterial spin labeling (ASL) to quantify cerebral blood flow (CBF) and found that 300-mL intragastric loads of xylitol, but not erythritol, increased CBF in the hypothalamus, whereas 75 g of glucose had the opposite effect. A recent study in young adults showed that, among those with a healthy weight, a sucrose-sweetened drink decreased lateral hypothalamus blood flow more than a sucralose-sweetened drink, but not more than water [31]. These findings add to the notion that different kinds of sweeteners can have differential effects on the brain although the impact of this on appetite regulation has not been elucidated.

Beverages are a predominant source of added sugar and caloric intake [[32], [33]]. Therefore, LNCSs are widely used to prevent increased energy intake from beverages, while still providing the hedonic experience of sweet taste. Previous studies have used different sweetener loads, usually administered as simple solutions in water that are ingested or infused. Next to classical carbonated soft drinks, flavored waters have been introduced as a less sweet alternative to sweet beverages, and their market is growing [34]. For research on sweeteners, they may constitute a more palatable and ecologically relevant alternative for simple solutions [25]. The aim of the current study was to determine the changes in regional brain activity, blood glucose and insulin concentrations, and gastric emptying in response to the ingestion of different flavored waters in individuals with healthy body weight. Beverages were sweetened with either the nutritive sugar sucrose or the LNCS such as sucralose, stevia extract, monk fruit extract, or the sugar replacement allulose (combined with stevia extract). Primary a priori regions of interest (ROIs) included the hypothalamus, a key homeostatic region sensitive to energy intake, and dopaminergic brain reward-related regions in the ventral striatum and midbrain responsive to food cues and energy intake [nucleus accumbens (NAcc), ventral tegmental area (VTA), substantia nigra (SN)]. The main hypothesis was that the sugar sucrose will elicit decreased CBF in brain areas related to food intake regulation and reward at 30 min, whereas equisweet low or nonnutritive sweeteners and water will not, due to their lack of, or lower, energy content. In addition, we examined whether there is a response in these brain areas at 5 min after ingestion, hypothesizing that this would be driven by the oral sweetness exposure.

## Methods

### Study design

This study was a partially double-blind, randomized, crossover trial in which healthy participants underwent brain imaging (fMRI), gastric MRI, and blood sampling at baseline and after consumption of water or a sweet flavored water. Participants and investigators were blinded for the treatment allocation until data analysis was complete with the exception of the water treatment, which was identifiable because of the lack of flavor and blood sampling. The primary outcome was CBF in a priori defined homeostatic and reward-related brain ROIs (hypothalamus, ventral striatum, and dopaminergic midbrain). Secondary outcomes were seed-based functional connectivity (resting-state fMRI), plasma glucose and insulin concentrations, and gastric content volume (GCV). Functional connectivity results will be reported elsewhere. Primary analyses were carried out fully blinded. In addition, subjective ratings of hunger, fullness, desire to eat, thirst, bloating, and well-being were collected. The study procedures were approved by an accredited Medical Research Ethics Committee (MREC Oost-Nederland; NL81742.091.22) in accordance with the Helsinki Declaration of 1975 as revised in 2013. The study was registered with clinicaltrials.gov under number NCT05575687 in October 2022. All participants provided informed consent.

### Sample size estimation

The sample size estimation was based on data from the hypothalamus because this region has been investigated the most with functional neuroimaging. Many fMRI studies have shown that the BOLD signal in the hypothalamus decreases after the ingestion of sugars, but not water [[22], [23], [24], [25], [26]]. In addition, there was a previous fMRI study [30] that used a similar intervention and primary outcome measurement (CBF) as used in this study. In line with the BOLD fMRI studies done, this study observed a lower regional CBF after ingestion of 75 g of glucose, compared with water. The mean difference was ∼2.4 mL/100 g/min. It is also known that the decrease in hypothalamic activity after glucose ingestion is dose dependent [22]. Accordingly, in the current study, the detectable difference in the hypothalamus between the 25-g sugar-containing drink and water was estimated to be 2.4/3 = 0.8 mL/100g/min. The SD was assumed to be of similar magnitude (0.8 mL/100g/min). To establish whether treatment effects differ from those in the water control condition, 5 treatments would be compared with water, as the primary analysis to show any sweetener’s effect. To account for multiple testings, we applied a Bonferroni correction and thus used an α of 0.05/5 = 0.01. Using the calculator for crossover studies at http://hedwig.mgh.harvard.edu/sample_size/js/js_crossover_quant.html, it was estimated that 30 datasets would be sufficient for 85% power of detecting a lower CBF in the hypothalamus. We also conducted a more advanced sample size estimation using version 2 of the GLIMMPSE online tool for mixed models at https://samplesizeshop.org/glimmpse-power-software [35]. This estimation showed that 30 datasets would be sufficient to detect a time × treatment interaction effect using a corrected threshold of *P* = 0.01, with powers ranging from 81% to 91% depending on the assumptions regarding mean effect size and variability (set as earlier and ±20%) and the type of test used (Hotelling Lawley Trace or Greenhouse-Geisser corrected). The within-treatment change in activity from baseline was expected to be larger than the difference between treatments [26]. Therefore, this sample size would also allow us to observe within-treatment changes in regional CBF, even if these are smaller than seen after sucrose, which was expected to have the strongest effect.

### Participants

Healthy (self-reported), right-handed individuals aged 18 to 30 years with a BMI (in kg/m^2^) of >18.5 and <25, with a sufficient blood hemoglobin concentration (females >7.5 g/dL; males >8.5 g/dL), and having antecubital veins suitable for blood sampling via a catheter were recruited. Only right-handed individuals were recruited because of possible brain differences between right- and left-handed individuals. Exclusion criteria were as follows: having disturbances of glucose metabolism such as having prediabetes or diabetes; Use of medication that could influence study results including insulin/metformin/proton pump inhibitors, antacids, antidepressants; allergy or intolerance for any of the study products/compounds (sucrose, sucralose, stevia extract, allulose, and monk fruit extract); being a regular smoker (smoking >1 cigarette or e-cigarette with nicotine per day); drinking >14 glasses of alcohol a week; having genetic, psychiatric or neurological diseases affecting the brain; having a gastric disorder or regular gastric complaints such as heart burn (more than once per week); having a renal or hepatic disease; using recreational drugs more than once per week (eg marijuana and laughing gas); donated blood in the past 2 months; being pregnant, lactating, or planning on becoming pregnant during the study; currently following or having followed a calorie-restricted diet in the past 2 mo; having a contraindication to MRI scanning (including but not limited to pacemakers and defibrillators, ferromagnetic implants, and claustrophobia).

Participants were recruited between January and December 2023 via digital advertisements (e-mail and social media) and invited for an online information session where they could ask questions. Those who appeared eligible and were willing to participate were invited for a screening session. During this session, they signed informed consent, filled in the inclusion questionnaire, and practiced drinking in a supine position in a mock MRI scanner by consuming 250 mL of water through a tube, similar to the test sessions; 35 participants were included in the study after screening. Of those 35, 2 were unresponsive and never scheduled for a test session, 2 resigned after 2 test sessions, and 1 resigned after 1 test session. Finally, 30 participants (15 females/15 males; age: 22.7 ± 2.3 years; BMI: 22.2 ± 1.7) completed 6 test sessions (Supplementary Figure 1) from 3 February, 2023, until 15 March, 2024.

### Treatments

The treatment consisted of the ingestion of 500 mL of water or 1 of 5 equisweet lemon-lime flavored waters sweetened with sucrose (25 g, 97 kcal), sucralose, stevia extract, monk fruit extract, or allulose+stevia extract. This amount of sucrose is realistic for this beverage category and has been shown to elicit a detectable glycemic response at 30 min [36,37]. The water control was not flavored because the flavor could not be matched to that of the flavored waters due to the absence of sweetness. Allulose (25 g in 500 mL; 5%; 5–10 kcal) was used in combination with stevia extract following the rationale that *1*) in commercial application, allulose does not usually fully replace sucrose in beverages but is used in combination with other sweeteners, stevia extracts in particular, to reach higher sugar reduction levels, improve taste quality, and reduce overall sweetener costs, and *2*) this dose will safeguard gastrointestinal tolerance: allulose is considered safe for human consumption up to 24 to 36 g (0.4–0.6 g/kg/d for a 60-kg individual) when consumed in 1 sitting, with a recommended maximal single dose where no gastrointestinal side effects are observed of 25 g [38,39].

The drinks were made by the Tate & Lyle Innovation Centre and provided in 500-mL pasteurized servings in plastic bottles labeled with a letter (A–F) for blinding. All drinks looked the same, like water (clear liquid). Detailed composition can be found in Supplementary Table 1. Treatment orders were determined by a research assistant by randomly sampling 6 sequential numbers (representing the 5 drinks) 40 times with the base function *sample* in R (R Core Team), including the *prob* (ie probability) argument to enforce a balanced distribution. By applying equal sampling probabilities, it was ensured that each drink appeared with similar frequency across all 40 orders and in each position within the sequence.

The drinks were consumed at room temperature, and participants were instructed to finish within 5 minutes, which they all did. The flavored drinks are briefly referred to by the sweetener used.

### Study procedures

**FIGURE 1:**
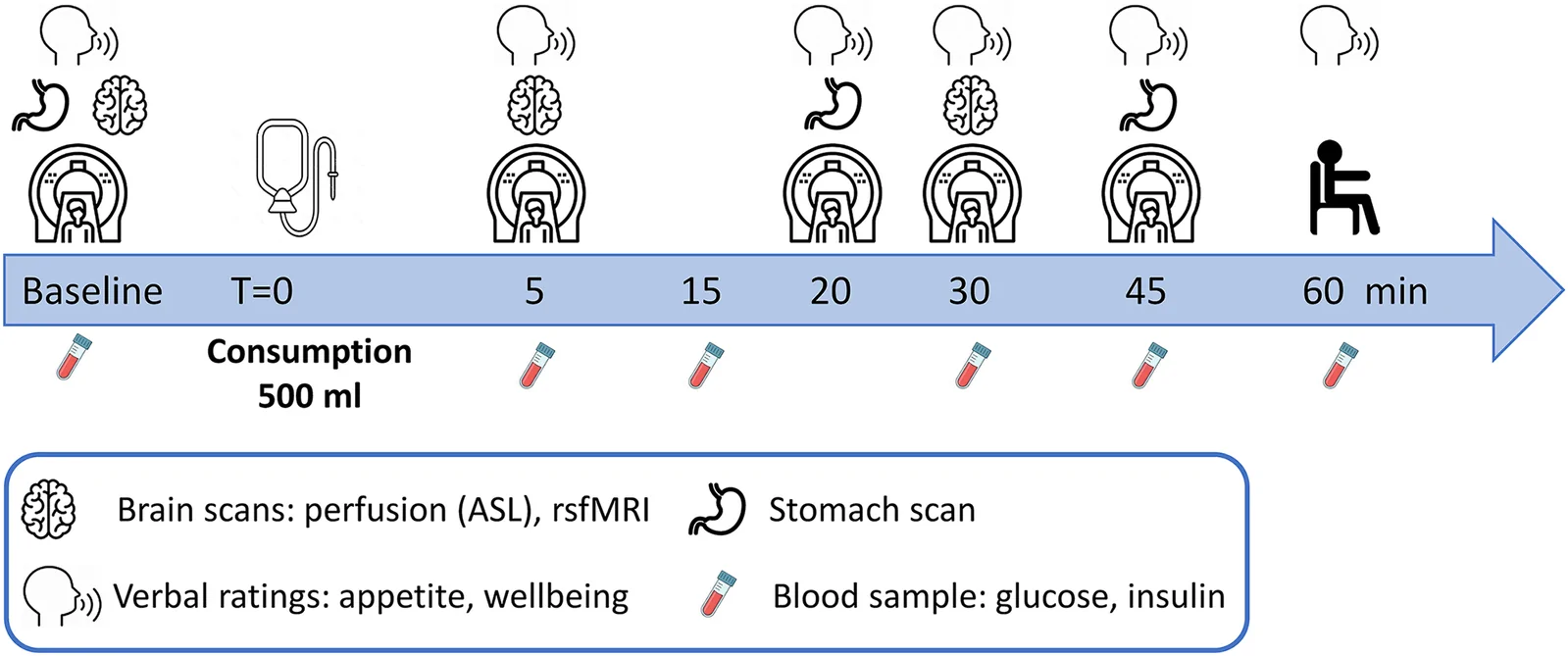
Overview of a test day. For water treatment, there was no blood sampling. *T* = 0 is the start of consumption from a tube connected to an irrigation bag. The MRI scanner icon indicates that MRI measurements were done, as specified earlier. At *T* = 60 min, the final measurements were done outside the scanner room while participants were seated. ASL, arterial spin labeling; MRI, magnetic resonance imaging; rsfMRI, resting-state functional MRI.

Study procedures are summarized in Figure 1. Participants were instructed to consume the same evening meal before each visit, to refrain from alcohol consumption that evening, and to fast from 22:00 onward (no food or drinks except water). Drinking water was allowed up to 90 min prior to their visit. Upon arrival at Hospital Gelderse Vallei (Ede, the Netherlands), a cannula was placed in an antecubital vein, and a blood sample was taken in case the treatment was a sweet drink. Subsequently, baseline MRI scans were performed and verbal subjective ratings [40] were obtained regarding hunger, fullness, desire to eat, bloating, well-being and thirst (“How hungry are you?,” “How full do you feel?,” “How much do you feel like eating?,” “To what extent do you feel bloated?,” “How well do you feel?,” “How thirsty are you?,” 100-unit scale with 0 = not at all and 100 = very much). After this, participants consumed 1 of the drinks in a supine position through a drinking tube connected to a standard irrigation bag, which was positioned at a height that required participants to actively draw the fluid through the tubing, similar to drinking through a straw. After the first sip, they verbally scored liking and sweetness on a similar 100-unit scale as the other ratings (“How tasty do you find the drink?” and “How sweet do you find the drink?”). All participants finished their drinks within 5 min.

After drinking, the *T* = 5 perfusion scan was made, followed by the resting-state fMRI scan and the *T* = 20 stomach scan. At *T* = 30, the brain scans were repeated, followed by another stomach scan at *T* = 45. After this, the participant was taken out of the scanner and seated. Except for water treatment days, postconsumption blood samples were taken at 5, 15, 30, 45 and 60 min. Appetite and well-being ratings were collected at 20, 30, 45 and 60 min. After this, participants were offered a takeaway breakfast.

The time between consecutive visits as per the study protocol was ≥3 d. In reality, it varied between 5 and 43 d, with the median per participant ranging from 6 to 15 d.

### MRI scanning

Participants were scanned head-first in a supine position with the use of a 3-Tesla Philips Ingenia Elition X MRI scanner, equipped with a dStream torso coil and a 32-channel head coil. The following scans were made:

• Stomach scan (18 s): a 2D turbo spin echo sequence (thrirty-three 5-mm slices; 1.4-mm gap; acquired in-plane voxel size, 1.8 × 1.8 mm, reconstructed to 1 × 1 mm; repetition time, 550 ms; echo time, 80 ms; flip angle, 90°; field of view, 400 × 336 × 210 mm; SENSE factor anterior-posterior, 2.5) was used with breath hold command on expiration to fixate the position of the diaphragm and the stomach.
• Anatomical brain scan (4 min), obtained at baseline: a high-resolution T_1_-weighted 3D fast field echo anatomical scan (repetition time, 9.8 ms; echo time, 4.5 ms; flip angle, 8°; field of view, 256 × 243 × 180 mm; 450 sagittal slices; scanning voxel size, 0.8 × 0.8 × 0.8 mm; reconstruction voxel size, 0.4 × 0.4 × 0.4 mm).
• Perfusion scan (5 min 20 s): to determine regional CBF, pseudocontinuous ASL [41] was performed with a segmented 3D readout using gradient and spin echo (GRASE) [42]. In total, 9 pairs of label and control images were acquired in anterior-posterior phase-encoding (PE) direction including 2 M0 images (labeling distance, 90 mm; postlabel delay, 1800 ms; repetition time, 4148 ms; echo time, 12 ms; flip angle, 90°; field of view, 240 × 240 × 102 mm; 17 axial slices; acquired voxel size, 3.74 × 4 × 6 mm; reconstructed voxel size, 3 × 3 × 6 mm). After each measurement, 2 additional M0 images were acquired with reversed PE direction (posterior-anterior).

### MRI image analysis

#### Cerebral blood flow

First, images were converted from DICOM to Nifti using dcm2niiX version v1.0.20020720. The T_1_-weighted images were used for segmentation, bias correction, and normalization of the anatomy using fsl_anat. The ASL data were analyzed using FSL 6.0.6.4 and Bayesian Inference for Arterial Spin Labeling (BASIL) tools. First, motion correction was performed for each series (mcflirt). Thereafter, distortion correction was carried out with the M0 images in different PE directions, and the correction was applied to the ASL images (topup). For calibration into absolute physiological units, voxel-wise mode was selected. Moreover, we chose “adaptive spatial regularization on performance” to reduce noise in the CBF map. Finally, partial volume correction was applied to restrict the analyses to gray matter, and the images were smoothed with an isotropic Gaussian kernel (full width at half maximum: 6 mm). For ROI analyses, we used the flag–region analysis that allowed extraction of mean CBF values from regions taken from the Harvard–Oxford cortical and subcortical atlases. Mean CBF values were extracted from homeostatic and reward-related brain areas of interest (Supplementary Figure 2): the hypothalamus, NAcc (ventral striatum), and VTA and SN (dopaminergic midbrain). The hypothalamus ROI was based on the Neudorfer atlas [43]. The NAcc was based on the Harvard–Oxford subcortical structural atlas as implemented in the FSL software. The VTA ROI was based on the probabilistic VTA atlas of Trutti et al. [44] accessible in the OpenScience Framework (https://osf.io/9pzj3). The SN ROI was taken from the automated anatomical labeling atlas 3 [45] (http://www.gin.cnrs.fr/en/tools/aal/). For all ROIs, CBF values were normalized by expressing them as a percentage of the mean global CBF [(ROI CBF/global CBF) × 100], which was calculated using all voxels that included ≥80% gray matter (derived from the structural segmentation).

#### Stomach scan image analysis

Total GCV was determined for each stomach scan with the help of an in-house software tool that performs a deep learning–based segmentation of the stomach contents based on an nnU-Net framework [46]. The resulting mask images were checked and, where necessary, manually corrected, with the use of the ITK-SNAP program [47] (http://www.itksnap.org/). An example of a stomach scan time series for all treatments is shown in Supplementary Figure 3.

### Blood sample collection and analysis

Blood samples were drawn from the cannula into sodium fluoride and lithium heparin tubes. After collection, all tubes were centrifuged at 1300 × *g* for 10 min at 20°C, to obtain blood plasma. Blood plasma aliquots were stored at −80°C until they were analyzed in bulk by a clinical chemistry laboratory (Ziekenhuis Gelderse Vallei, Ede, the Netherlands). To determine glucose concentration, the sodium fluoride plasma samples were processed using the Atellica CH Glucose Hexokinase_3 (GluH_3) assay kit and quantified using the Atellica CH analyzer (Siemens Healthineers). The detection range was 0.2 to 39 mmol/L, and the maximum intra-assay coefficient of variation was 4.5%. There were no glucose concentrations outside the detection range. The lithium heparin plasma samples were processed, and insulin was quantified using a solid-phase, enzyme-labeled chemiluminescent immunometric assay (IMMULITE 1000 Insulin) and an Atellica IM Analyzer (Siemens Healthineers). The detection range was 2 to 300 μIU/L, and interassay coefficient of variations ranged from 3.3% to 19.1%. Values below the detection limit were set at 1 μIU/L (lower limit/2) as a biologically plausible low value for healthy normal-weight young adults. There were no insulin concentrations above the detection limit. The Atellica analyzers are validated for single-replicate clinical measurements; no duplicates were analyzed. Because 339 of 900 concentrations (37.7%) were below the detection limit, the data were log-transformed before analysis.

### Statistical analysis

Statistical analyses were performed using RStudio (version 4.4.1, 2024.04.0, Posit Software) and the R packages dplyr (1.1.4), emmeans (1.10.3), ggeffects (1.7.0), multcomp (1.4-25), and nlme (3.1-164) on data from the 30 participants who completed 6 test sessions. Missing values were not imputed. Primary analyses were performed blinded for treatment. Blinding was not complete because the water treatment was the only nonsweet drink and no blood samples were taken during visits involving this treatment.

Drink perception (liking and sweetness) was compared between treatments with the use of linear mixed models (lme), with treatment as a fixed factor, treatment order as a categorical covariate, and participant as a random factor. For the primary ROI CBF analysis on the hypothalamus and reward-related ROIs [ventral striatum (NAcc) and midbrain (VTA and SN)], we used linear mixed models (lme), with participant as a random factor (random intercept) and the treatment effect of interest (ΔCBF) as the dependent variable. Fixed effects included treatment, and global CBF, treatment order, and sex were included as covariates to examine *1*) each drink’s change in CBF from baseline at *T* = 30 min (treatment effect) and *2*) each drink’s change in CBF at *T* = 30 min compared with that of sucrose (main treatment difference). In addition, we tested *3*) each sweet drink’s change from baseline CBF at *T* = 5 min compared with that of water (effect of sweetness), and *4*) each drink’s change in CBF from *T* = 5 to *T* = 30 compared with that of sucrose).

Furthermore, an exploratory voxel-wise whole-brain analysis was performed in SPM12. For this, the CBF maps were entered into a 6 × 3 full factorial model with treatment (6 levels) and timepoint (3 levels: baseline, *T* = 5 min, and *T* = 30 min) as within-subject factors. Both factors were specified as dependent (repeated-measures), and equal variance (pooled error variance with common error term for all cells of the design) was assumed, as in a classical repeated measurement analysis of variance. Global CBF, treatment order, and sex were added as covariates [global CBF was mean centered; treatment order (1–6) and sex (0 or 1) were not centered]. There were 2 missing datapoints, 1 for allulose+stevia and 1 for water.

Subsequently, we created contrasts for the main effect of time (i.e., collapsed over all conditions) between T5 and baseline and between T30 and baseline to examine changes in CBF over time. Moreover, we computed interactions between sucrose as a reference condition and the other conditions separately for T5 compared with baseline, T30 compared with baseline, and T30 compared with T5 to test for differential time effects between the treatments. A primary statistical threshold of uncorrected *P* < 0.001 and a familywise error (FWE)-corrected *P* < 0.05 for multiple comparisons at the cluster level was applied. Small volume correction (SVC) was applied for the a priori primary ROIs (hypothalamus, NAcc, VTA, and SN) and for additional subcortical ROIs involved in food reward processing and the regulation of food intake: the basal ganglia (putamen, caudate, and globus pallidus) and the amygdala [48,49]. A combined ROI mask of these regions was created for SVC.

For the secondary outcomes—glucose, insulin, GCV, and subjective ratings—the main effect of treatment and treatment × time interactions were examined with linear mixed models (lme), with the treatment × time interaction as a fixed factor (including all time points excluding the baseline measurement), baseline values for each visit as a covariate, treatment order as a categorical covariate, and participant as a random factor (random intercept). For insulin, log-transformed concentrations were used because of the skewed distribution. Contrast estimates were backtransformed to obtain difference ratios. As a sensitivity analysis, we repeated the insulin analysis without log-transforming and with using 2, i.e., the lower limit of detection, instead of 1. Both these approaches did not change the pattern of the results.

For the ROI CBF and GCV analyses, sex was added as a covariate because sex can affect brain function [50], including perfusion [51] and digestion [52]. Benjamini–Hochberg false discovery rate (FDR)-corrected *P* values were calculated for all pairwise comparisons within each model.

## Results

### Drink perception

Data are shown in Supplementary Figure 4. Liking did not differ among the sweet drinks except for monk fruit, which was liked modestly less than sucrose (difference: 9.49 ± 3.73; *P*_FDR_ = 0.036). All drinks except monk fruit (difference: 7.30 ± 3.73; *P*_FDR_ = 0.13) were liked significantly more than water (all differences > 12.7 ± 3.7; all *P*_FDR_ < 0.003). Perceived sweetness of all drinks was greater than that of water (all differences > 44 ± 3.5; all *P*_FDR_ < 0.001). Sweetness did not differ significantly among most flavored drinks (all *P*_FDR_ > 0.2), with the exception of monk fruit and allulose+stevia, which were rated modestly less sweet than sucrose (difference for both: 9.4 ± 3.4; *P*_FDR_ = 0.017).

### CBF response in ROIs

**FIGURE 2:**
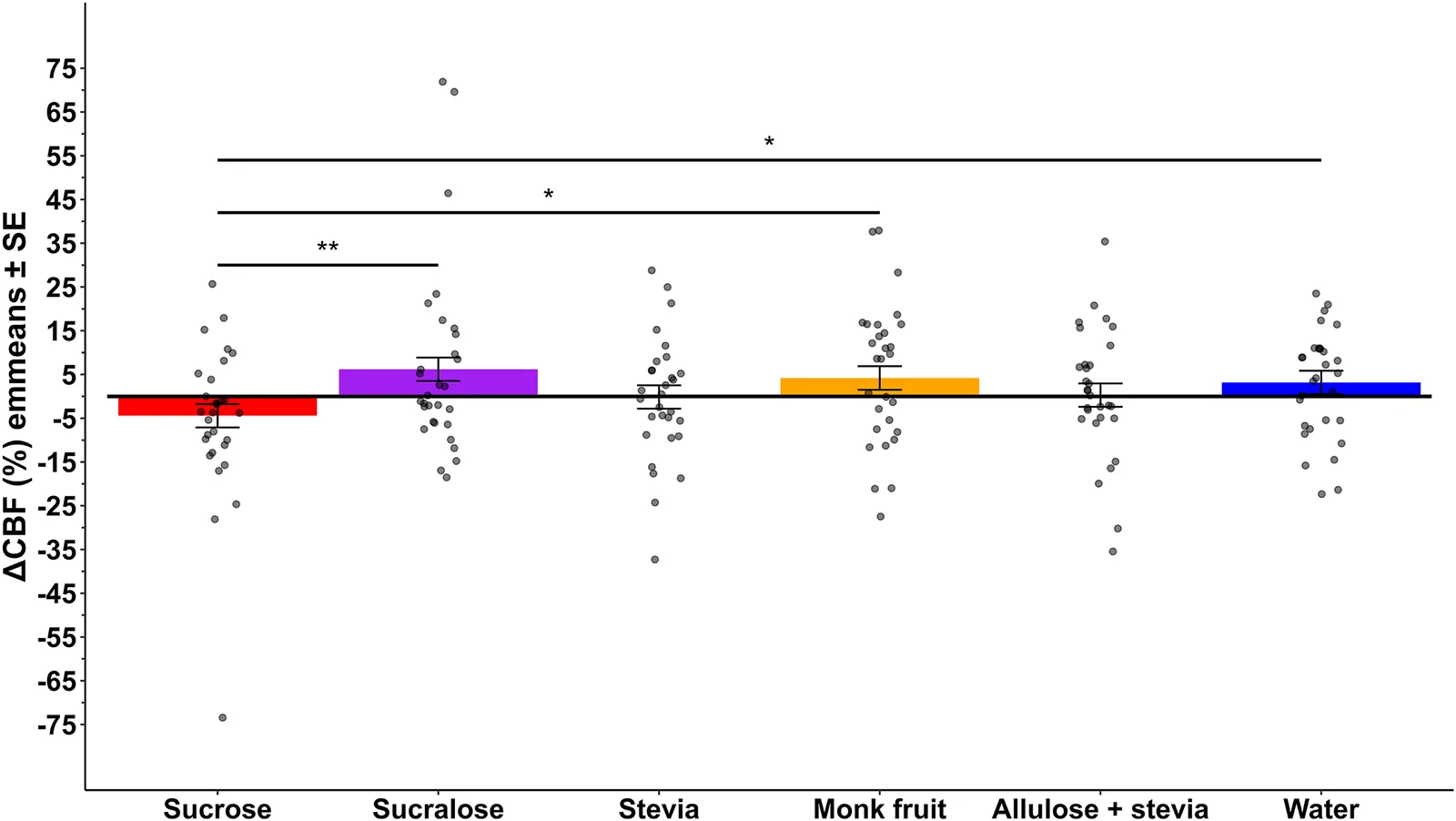
Estimated marginal mean normalized change in cerebral blood flow (CBF; % of global CBF) ± SE from baseline at *T* = 30 min in the ventral tegmental area region of interest for all treatments, based on a linear mixed model, with baseline, order of treatment, and sex as covariates. Data points represent the unadjusted individual values. ΔCBF for all treatments was compared with that of sucrose. ∗*P*_FDR_ < 0.05.
• In addition, we tested each sweet drink’s change from baseline CBF at *T* = 5 min compared with that of water (effect of sweetness): None of the sweet drink’s change in ROI CBF at *T* = 5 differed from that of water (Supplementary Table 5).
• Finally, we assessed each drink’s change in CBF from *T* = 5 to *T* = 30 compared with that of sucrose: in the VTA, all drinks showed a relative increase in CBF compared with sucrose (Figure 3, Supplementary Table 6) (difference for sucralose: 9.69 ± 4.53; *P*_FDR_ = 0.034; stevia: 10.40 ± 4.54; *P*_FDR_ = 0.034; monk fruit: 10.13 ± 4.58; *P*_FDR_ = 0.034; allulose+stevia: 9.73 ± 4.54; *P*_FDR_ = 0.034; water: 14.45 ± 4.59; *P*_FDR_ = 0.010).

Raw global and ROI CBF are shown in Supplementary Table 2, showing the regional stability of the measurements. Drink-induced changes in CBF in a priori homeostatic (hypothalamus) and reward-related [ventral striatum (NAcc) and midbrain (VTA and SN)] ROIs were assessed as follows:

• Change from baseline CBF at *T* = 30 min for each treatment and primary ROI: in the hypothalamus, NAcc, SN, and VTA, none of the drinks induced a significant change in CBF from baseline (Supplementary Table 3).
• Each drink’s change in CBF at *T* = 30 min compared with that of sucrose (main treatment difference): in the hypothalamus, NAcc, and SN, the change in CBF at *T* = 30 did not differ from that of sucrose (Supplementary Table 4). In the VTA, monk fruit, sucralose, and water showed a greater change from baseline than sucrose (Figure 2) (difference for monk fruit: 8.36 ± 3.29; *P*_FDR_ = 0.024; sucralose: 10.62 ± 3.26; *P*_FDR_ = 0.007; water: 7.61 ± 3.27; *P*_FDR_ = 0.036).

**FIGURE 3:**
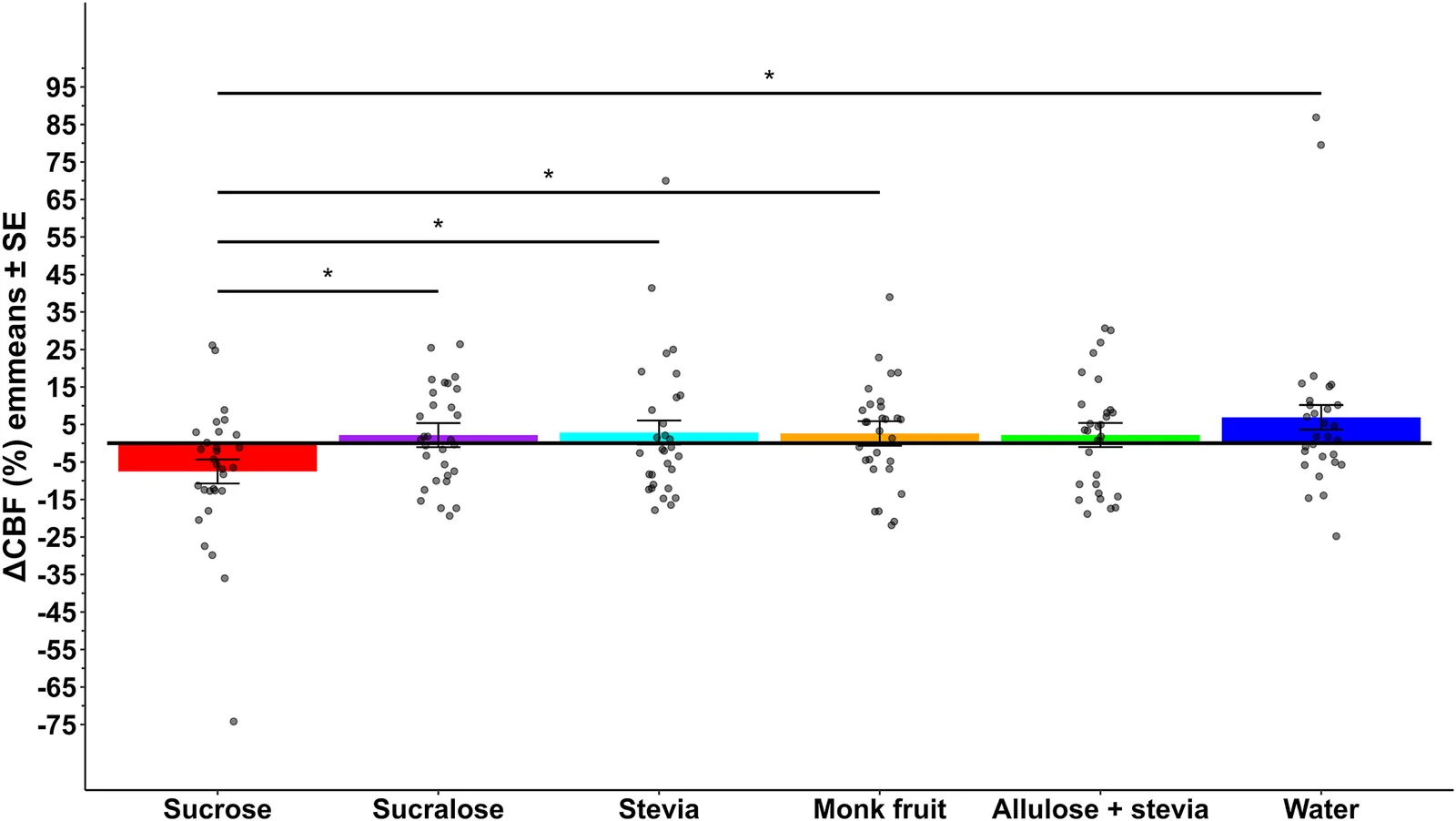
Estimated marginal mean normalized change in cerebral blood flow (CBF; % of global CBF) ± SE from *T* = 5 to *T* = 30 min in the ventral tegmental area region of interest for all treatments, based on a linear mixed model, with baseline, order of treatment, and sex as covariates. Data points represent the unadjusted individual values. ΔCBF for all treatments was compared with that of sucrose. ∗*P*_FDR_ < 0.05.

### Exploratory whole-brain analysis (LNCS compared with sucrose)

Results are shown in Table 1 and Supplementary Figure 5. Compared with baseline, there was an increase in CBF across conditions in the putamen at *T* = 5 min (peak *P*_FWE_ < 0.05, small volume corrected). This effect was still evident, albeit less pronounced at *T* = 30 min. Furthermore, at *T* = 5 min, CBF was decreased in the superior temporal gyrus and fusiform gyrus. At *T* = 30 min, superior temporal gyrus CBF was decreased (for all results: *P*_FWE_ < 0.05 cluster-level corrected).

**TABLE 1:** Results of the exploratory whole-brain CBF analysis (full factorial model)^1^

Brain region	MNI coordinate (x, y, z)	Peak *T* value; cluster size (k)	Cluster-level *P*_FWE_/peak *P*_FWE_ (SVC)
Time effects across all 6 treatments			
T5 > baseline			
Putamen R	36, −4, 2	5.04; 166	0.00035 (SVC)
Putamen L	−26, 4, −8	4.29; 123	0.008 (SVC)
Baseline > T5			
Fusiform gyrus R	−24, −50, −18	6.96; 23,519	<0.001
Superior temporal gyrus L	−50, −8, −8	5.94; 965	<0.001
T30 > Baseline			
Putamen R	36, −4, 0	4.30; 238	0.008 (SVC)
Putamen L	−20, 8, −8	3.83; 49	0.044 (SVC)
Caudate R	16, 4, 14	4.16; 83	0.013 (SVC)
Baseline > T30			
Superior temporal gyrus R	44, −16, −4	7.20; 2486	<0.001
Superior temporal gyrus L	−44, −22, 0	6.86; 1617	<0.001
Vermis	0, −56, −22	6.94; 1318	<0.001
Middle cingulate gyrus L	−4, −24, 44	4.03; 666	0.001
Interaction effects treatment × timepoint			
Sucrose and allulose+stevia × time interaction^2^			
Amygdala L	−20, −2, −14	4.52; 51	0.003 (SVC)
Sucrose and stevia × time interaction^3^			
Putamen L	−30, −6, −4	4.27; 105	0.009 (SVC)
Caudate R	18, 22, 2	3.96; 30	0.028 (SVC)
Sucrose and water × time interaction^4^			
Putamen R	26, 10, −10	4.17; 32	0.013 (SVC)
Sucrose and water × time interaction^5^			
Putamen R	22, 18, 0	3.92; 42	0.032 (SVC)

Abbreviations: CBF, cerebral blood flow; L, left hemisphere; R, right hemisphere; SVC, small volume-corrected peak *P*_FWE_ value for the mentioned brain region (a priori region of interest).

1 Model with global CBF, sex, and treatment order as covariates.

2 Contrast: [Sucrose(T30) − Sucrose(T5) + Allulose and stevia(T30) − Allulose and stevia(T5)].

3 Contrast: [Sucrose(T30) − Sucrose(baseline) + Stevia(T30) − Stevia(baseline)].

4 Contrast: [Sucrose(T30) − Sucrose(T5) + Water(T30) − Water(T5)].

5 Contrast: [Sucrose(baseline) − Sucrose(T30) − Water(baseline) + Water(T30)].

There were 4 treatment by timepoint interactions for LNCS and sucrose (all peak *P*_FWE_ < 0.05, small volume corrected; illustrated in Supplementary Figure 4). In the left amygdala, the change in CBF from *T* = 5 to *T* = 30 differed between sucrose (decrease) and allulose+stevia (increase). Similarly, in the left putamen and right caudate, the change in CBF from baseline to *T* = 30 differed between sucrose (decrease) and stevia (increase). In the right putamen, the changes from baseline to T5 and *T* = 30 differed between sucrose (decrease) and water (increase).

### Glucose and insulin

**FIGURE 4:**
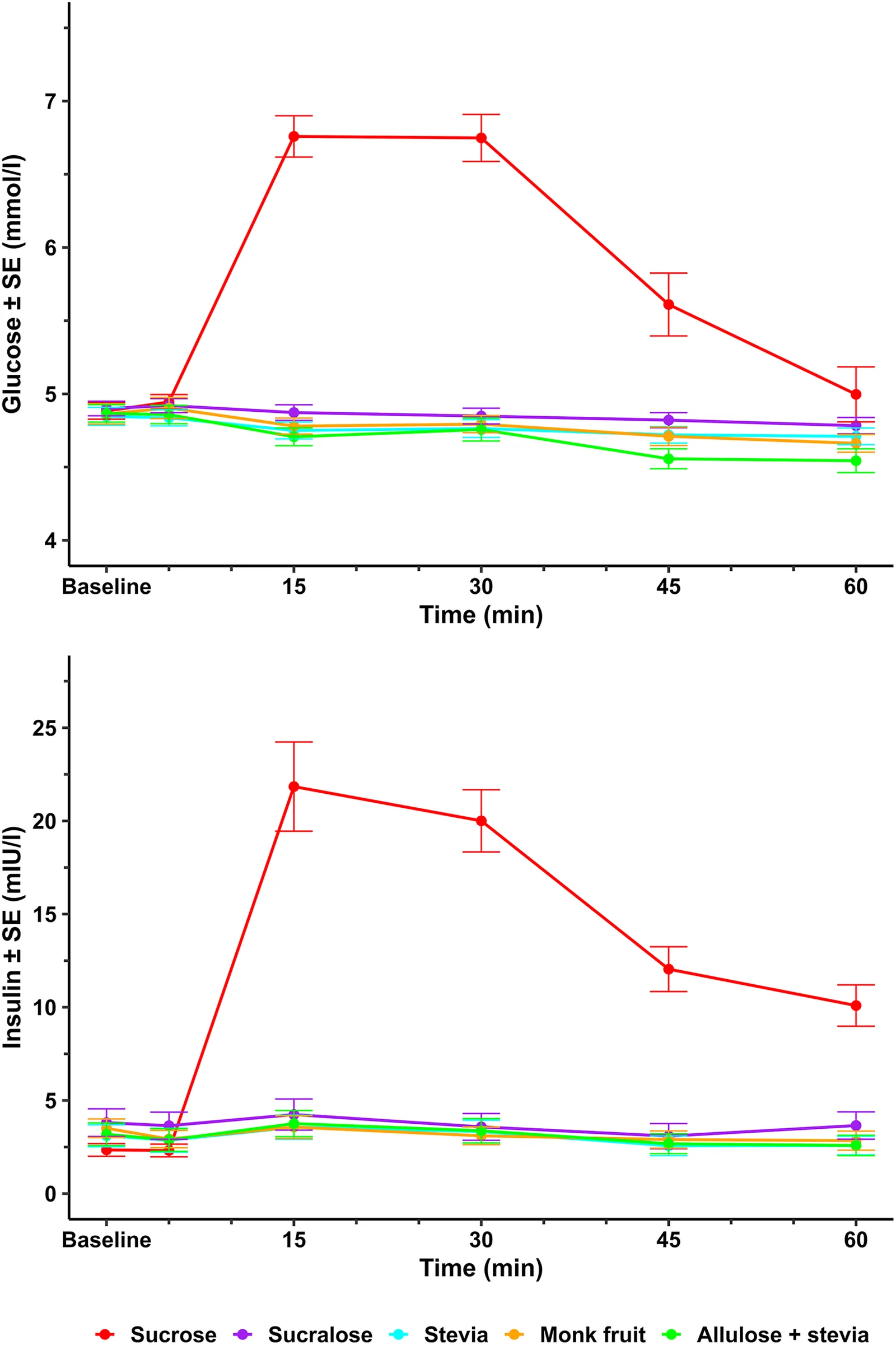
Mean ± SE glucose (top) and insulin (bottom) concentrations over time for all treatments.

Results are shown in Figure 4. For glucose, there was a main effect of time, treatment, and a treatment × time interaction (all *P* < 0.001). At *T* = 5, there were no differences between the drinks. At *T* = 15, *T* = 30, and *T* = 45 min, all drinks differed from sucrose (all *P*_FDR_ < 0.001; largest difference: 2.03 ± 0.11 at *T* = 15 min; smallest difference: 0.79 ± 0.11 at *T* = 45). At *T* = 45 min, there was higher glucose for sucralose than for allulose+stevia (difference: 0.25 ± 0.11; *P*_FDR_ = 0.046). At *T* = 60 min, allulose+stevia and monk fruit (*P*_FDR_ = 0.021) still had slightly lower glucose than sucroseis (difference: 0.43 ± 0.11 *P*_FDR_ < 0.001; and 0.31 ± 0.11; *P*_FDR_ = 0.021, respectively); similarly stevia and sucralose tended to differ from sucrose (difference: 0.24 ± 0.11; *P*_FDR_ = 0.077; and 0.22 ± 0.11; *P*_FDR_ = 0.093, respectively).

For insulin, there was a main effect of time, treatment, and a treatment × time interaction (all *P* < 0.001). At *T* = 5, there were no differences between the drinks (all *P*_FDR_ = 0.89). For all later timepoints, insulin was higher for sucrose than for all other drinks (all *P*_FDR_ < 0.001; largest difference: 7.8- to 9.3-fold at *T* = 15 min; smallest difference: 4.2- to 5.3-fold at *T* = 60).

### Gastric content volume

**FIGURE 5:**
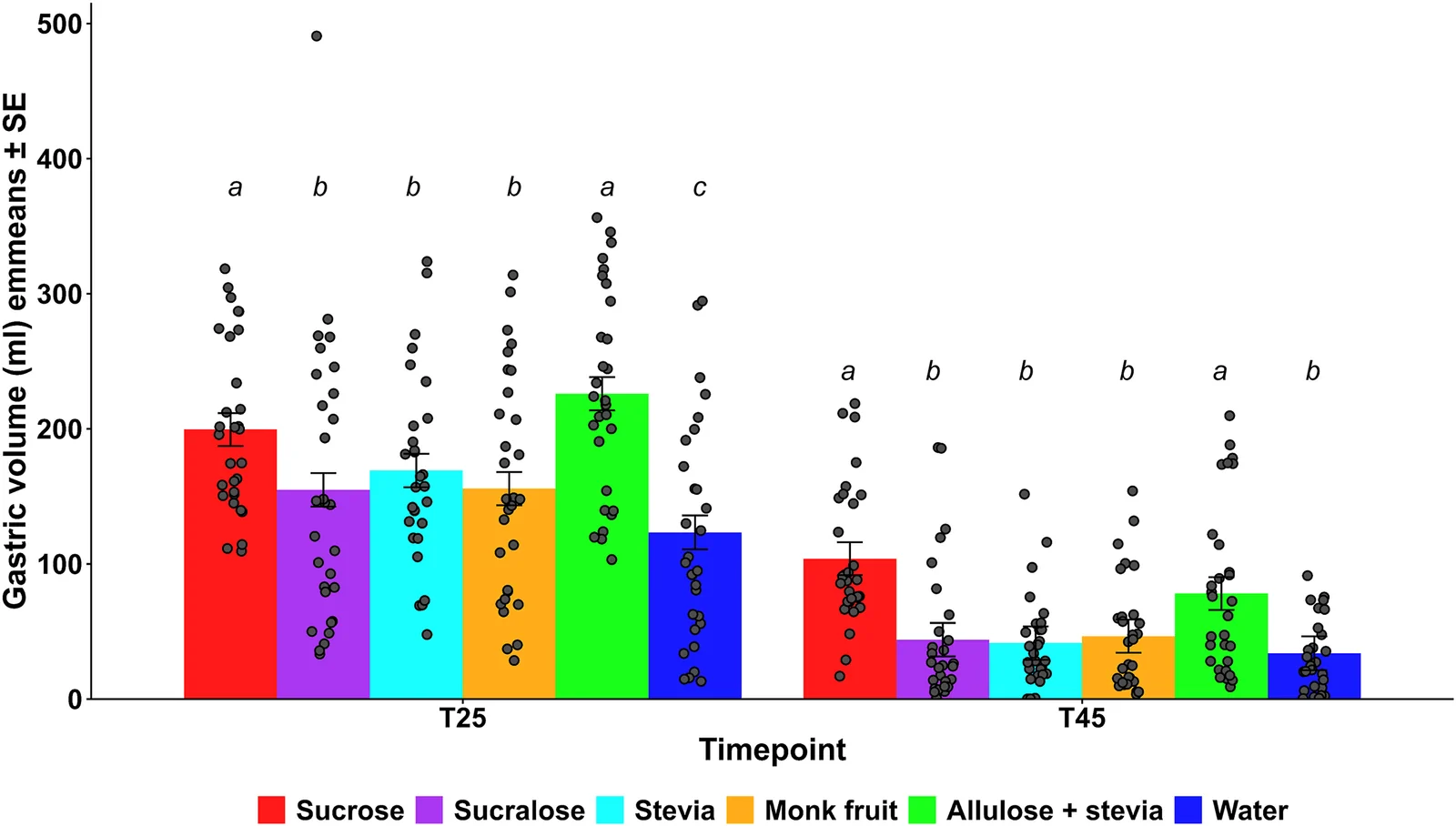
Estimated marginal means (emmeans) ± SE for gastric content volume over time based on the linear mixed model, with baseline and sex as covariates. Data points represent the unadjusted individual values. Different letters indicate that treatments differ significantly at a timepoint (*P*_FDR_ < 0.05). A plot of the unadjusted means can be found in Supplementary Figure 6.

Results are shown in Figure 5, Supplementary Figure 6 (unadjusted mean volumes). and Supplementary Table 7 (differences between treatments per timepoint). There was a main effect of treatment such that GCV was higher for sucrose and allulose+stevia than for the other drinks: time (both *P* < 0.001) and a treatment × time interaction (*P* = 0.020). At baseline, there were no differences between the drinks. At *T* = 25, sucrose had a greater GCV than all other treatments except for allulose+stevia (difference with—water: 76 ± 13; *P*_FDR_ < 0.001; sucralose: 45 ± 13; *P*_FDR_ = 0.002; monk fruit: 44 ± 13; *P*_FDR_ = 0.002; stevia: 30 ± 13; *P*_FDR_ = 0.028; allulose+stevia: −27 ± 13; *P*_FDR_ = 0.051). Allulose+stevia had a greater GCV than water (103 ± 13; *P*_FDR_ < 0.001) and all the other sweet drinks except for sucrose (difference with—sucralose: 71 ± 13; *P*_FDR_ < 0.001; monk fruit: 70 ± 13; *P*_FDR_ < 0.001; stevia: 57 ± 13; *P*_FDR_ < 0.001). At *T* = 45, sucrose had a larger GCV than all other treatments except for allulose+stevia (difference with—water: 70 ± 13; *P*_FDR_ < 0.001; sucralose: 60 ± 13; *P*_FDR_ < 0.001; monk fruit: 57 ± 13; *P*_FDR_ < 0.001; stevia: 62 ± 13; *P*_FDR_ < 0.001; allulose+stevia: 26 ± 13; *P*_FDR_ = 0.074). Allulose+stevia had a greater GCV than water (difference: 44 ± 13; *P*_FDR_ = 0.003), sucralose (difference: 34 ± 13; *P*_FDR_ = 0.020), stevia (difference: 37 ± 13; *P*_FDR_ = 0.013), and monk fruit (difference: 32 ± 13; *P*_FDR_ = 0.029).

### Subjective ratings

Data are shown in Supplementary Figure 7. For hunger, there was a main effect of treatment and time (both *P* < 0.001) but no interaction effect (*P* = 0.997). Hunger was lower for sucrose than for water (treatment difference: 6.71 ± 1.27; *P*_FDR_ < 0.001), stevia (difference: 4.07 ± 1.25; *P*_FDR_ = 0.004), and monk fruit (difference: 2.93 ± 1.26; *P*_FDR_ = 0.0498) and for sucralose than for water (difference: 4.95 ± 1.26; *P*_FDR_ < 0.001), allulose+stevia than for water (difference: 4.90 ± 1.28; *P*_FDR_ < 0.001), and monk fruit than for water (3.78 ± 1.25; *P*_FDR_ = 0.008).

For fullness, there were treatment (*P* = 0.003) and time (*P* < 0.001) effects but no interaction effect (*P* = 0.341). However, there were no significant treatment differences post hoc.

For desire to eat, there was a main effect of treatment and time (both *P* < 0.001) but no interaction effect (*P* = 0.962). Desire to eat was lower for all sweet drinks than for water (treatment differences, all *P*_FDR_ < 0.001—sucrose: 6.63 ± 1.39; sucralose: 6.59 ± 1.38; stevia: 5.47 ± 1.38; monk fruit: 5.09 ± 1.37; allulose+stevia: 5.06 ± 1.39).

Thirst declined after consumption but did not differ significantly between the treatments (*P* = 0.067). Bloating ratings were very low, ∼20, and showed no significant differences between treatments (all *P* > 0.063). Well-being was scored ∼70 and did not differ between treatments (*P* = 0.464).

## Discussion

We examined changes in CBF in response to the ingestion of flavored waters sweetened using a modest 25-g dose of the sugar sucrose or LNCS and water. In addition, changes in GCV, insulin and glucose concentrations (for all sweet treatments), and appetite were measured. Although no significant differences in hypothalamus blood flow were observed and appetite changes were similar, LNCS ingestion resulted in distinct brain and gastric responses compared with 25 g of sucrose. Drinks containing allulose+stevia, or stevia alone, altered activity in reward-related brain regions. The allulose+stevia drink also delayed gastric emptying to a degree comparable with the sucrose drink, whereas only the latter drink increased glucose and insulin concentrations.

### Brain responses

In the hypothalamus, the primary ROI, no differential treatment effects were found. This contrasts with previous studies reporting differences between glucose or fructose ingestion and water on hypothalamus activity using functional MRI—either CBF [28,30] or BOLD signal [26,29]. Unlike most previous studies, we used the disaccharide sucrose (ie glucose+fructose), which is commonly used in beverages in Western Europe. Page et al. [28] reported a decline in hypothalamus CBF after ingestion of 75 g of glucose, but not fructose, dissolved in flavored water. In a recent study in a relatively large sample (*n* = 75) with a broad BMI range, the effect of a 300-mL drink containing 75 g sucrose on hypothalamus blood flow did not differ from that of water [32]. Specifically, in individuals with healthy weight (*n* = 26), the decline in lateral hypothalamic blood flow was >75 g sucrose compared with sucralose but did not differ from water [32]. Van Opstal et al. [26] found a more delayed and smaller decrease in hypothalamus activity after ingestion of 50 g of fructose and sucrose compared with glucose ingestion and attribute this to the slower metabolization of fructose. Intragastric loads of the sugar alcohol xylitol (50 g in 300 mL of water), but not erythritol (75 g), increased CBF in the hypothalamus, whereas 75 g glucose in 300 mL of water had the opposite effect [30]. In line with these findings, dose-dependent BOLD signal decreases were reported in parts of the hypothalamus after ingestion of 25 and 75 g of glucose in 300 mL of water [22,53]. Although this was considered in our sample size estimation, the dose of 25 g of sucrose may have been too low to elicit a sustained reduction in hypothalamus activity (lower CBF). The overall effect of sucrose on the hypothalamus might be small, since fructose tends to increase hypothalamus CBF [28], potentially counteracting any glucose-related decrease.

In the VTA, a core hub for reward processing, ΔCBF was higher at 30 min for sucralose, monk fruit, and water than for sucrose. This aligns with the finding that VTA activity decreased with glucose but increased similarly for water and sucralose (sweetness matched to 50-g glucose) up to 22 min after ingestion [26]. This was proposed to be due to the absence of energy and possibly a reflection of lower satiety [26]. Likewise, in our study, a higher VTA response was identified at the later time point but not shortly after ingestion, pointing to the possible influence of satiety signaling on VTA activity rather than an acute effect of the sweet taste. The drinks with allulose+stevia and stevia did not show different ΔCBF in the VTA at 30 min, indicating differential responses based on the LNCS type. The VTA integrates homeostatic and reward signals to regulate the intake of palatable foods. Whether the differential effects on VTA activity of some LNCS drinks could affect eating behavior needs further investigation.

In the exploratory brain analysis, increased CBF was observed in the left putamen and amygdala for stevia and allulose+stevia. This is in line with previous studies showing differential striatal and amygdala activation and functional connectivity in response to different LNCS [19,30,54]. Further studies are needed to investigate the effects of different types of LNCS or combinations of sweeteners on reward processing.

In summary, we found differences in the effects of the ingestion of various sweeteners on brain regions involved in reward, motivation, and energy intake regulation in individuals with a healthy weight. However, the hypothalamus, the primary ROI, did not show the expected differential treatment effects observed in previous studies [23,26,28,32,54]. It should be noted that these studies differed in participants’ body weight ranges, as well as in the dosage and type of sweeteners used, and did not always adjust their CBF analyses for global CBF changes complicating direct comparisons with the current study. All sweet drinks in this study contained a lemon-lime flavor. Thus, comparisons with water could show effects of the flavor next to sweetness effects; however, the data show no indication of this.

### Glycemic responses, gastric emptying, and appetite

As expected, only sucrose resulted in increased blood glucose and insulin concentrations. The drink with 25 g of allulose+stevia, which is low-caloric because allulose provides 90% to 95% less energy than sucrose [55,56], showed a very small reduction in blood glucose, in the absence of any insulin effects. Although not deemed physiologically relevant, this observation is in line with studies that examined the glucose-lowering effects of allulose when coingested with a sugar [57].

Despite its low energy content, allulose+stevia slowed gastric emptying compared with water and the LNCS drinks, similar to sucrose. That drink was expected to show the slowest gastric emptying because of its caloric content. Unlike the other LNCSs, allulose was the only bulking sweetener used in a higher quantity, which provides a viscosity and mouthfeel similar to that of sucrose, which also has bulking properties. Allulose can induce glucagon-like peptide (GLP)-1 release, which in turn could inhibit gastric emptying. Teysseire et al. [58] found GLP-1, cholecystokinin, and peptide YY release after intragastric administration of 25 g of allulose in 300 mL of tap water. Despite the release of GLP-1 and other appetite-related hormones, they observed no significant effect on gastric emptying. When paired with lactisole, an inhibitor of the sweet taste receptor (T1R2/T1R3), similar results were obtained. This is in line with earlier work in which blocking the sweet taste receptors in the gastrointestinal tract with lactisole suppressed appetite-related hormone release but did not affect gastric emptying of a 75-g intragastric glucose load [59]. A key difference with our study is that we had orosensory exposure to sweetness, matched to that of 50 g/L sucrose, as well as a direct measurement of GCV. Exposure to sweetness in the absence of energy in the form of a diet soda can augment the GLP-1 response to subsequent ingestion of a 75-g glucose load [60]. We observed slower gastric emptying at *T* = 25 min for all LNCS drinks compared with water, whereas 20 min later, the GCV of the sweet drinks without energy was similar to that of water. This suggests a short-lived effect of sweetness alone on gastric emptying. Blocking oral sweet taste perception with lactisole resulted in somewhat slower gastric emptying of a 15% glucose solution, whereas emptying of an equisweet aspartame solution was unaffected [61]. Taken together, this suggests that postoral sweetness signaling can affect gastric emptying. Another factor that can affect gastric emptying is the osmolarity of the chyme, which is sensed by osmoreceptors in the duodenum. Nonisotonic duodenal contents slows down gastric emptying [[62], [63], [64]]. Hyperosmolar duodenal content lowers plasma ghrelin concentrations and augments cholecystokinin, peptide YY, and GLP-1 concentrations in healthy individuals [65]. In our study, the sucrose drink was hypotonic (146 mOsm/L) and allulose+stevia was isotonic (278 mOsm/L). Thus, the slower gastric emptying of allulose+stevia than of water (hypotonic) is likely not due to osmolarity differences. Taken together, this suggests that the slower gastric emptying of the allulose+stevia drink may have been driven by allulose-induced GLP-1 release. However, this did not decrease desire to eat more than seen for the other sweet drinks; all sweet drinks were associated with modestly lower desire to eat and hunger scores (4–6 points/100).

### Limitations

Our sample size estimation was based on available summary data [30] because of a lack of individual-level data or correlation estimates. This limited the extent to which within-subject and between-treatment correlations could be incorporated in the calculation. The study might have been underpowered. However, smaller effects than those elicited by our modest but ecologically valid 25-g sucrose dose would likely not be physiologically meaningful. Three participants who dropped out after only 1 or 2 visits were excluded from the analysis and replaced. In addition, because the randomization was not counterbalanced for order, we cannot entirely exclude potential effects of increasing familiarity with the study procedures, although treatment order was included as a covariate, assuming there are no exposure by order interaction effects. Results from our young healthy population cannot be generalized to other populations. We did not control for menstrual cycle phase. However, sex was used as a covariate where deemed appropriate, and in previous studies with young Dutch women, we established that a majority uses an intrauterine device or oral contraceptive pills. This leaves less room for potential cycle phase effects. Finally, the substantial number of statistical tests conducted overall increases risk of false-positive findings.

## Conclusions

In conclusion, various LNCS-flavored waters generally did not alter regional CBF or gastrointestinal responses compared with water ingestion in healthy normal-weight adults, although some differences in reward-related brain areas were noted compared with a 25-g sucrose drink. Some differential effects related to allulose+stevia and stevia ingestion were observed in brain regions involved in the regulation of food intake, warranting further exploration. Allulose+stevia delayed gastric emptying similar to an equisweet drink with sucrose, consistent with its ability to induce GLP-1 release. Overall, this suggests that certain nonnutritive sweeteners can have specific neural and gastrointestinal effects despite their minimal energy content. However, different LNCSs and water did not differ in their acute glycemic effects, and there were no differential effects on appetite of the different sweeteners. Further exploration of the specific neural and physiological effects of stevia extract and allulose is warranted.

## Author contributions

The authors’ responsibilities were as follows—PAMS, DR: designed the research; EO, PS, SM: conducted the research; PS, SK, RV: analyzed data and wrote the paper; HP, PS, RV, SK: interpreted the results; PS, SK had primary responsibility for final content; and all authors: provided feedback on the draft manuscript and read and approved the final manuscript.

## Data availability

Data described in the manuscript and analytic code will be made available for research use upon reasonable request to the corresponding author.

## Declaration of Generative AI and AI-assisted technologies in the writing process

The authors declare that no generative AI or AI-assisted technologies were used in the writing of this manuscript.

## Funding

This work was funded by Tate & Lyle PLC who was involved in the design of the study and assisted with writing of the paper (review of the draft paper and approval of the final version). There were no restrictions on the ability to publish the results of the study as laid down in the collaborative research agreement other than the right to reasonably withhold or redact any company confidential information relating to the research results within 3 months.

## Conflict of interest

PS is an Editor for *The American Journal of Clinical Nutrition* and played no role in the Journal’s evaluation of the manuscript. There other authors report no conflicts of interest.
