# Supplementary material for: Brain and physiological responses to flavored waters with different sweeteners: a randomized crossover study in healthy young adults
Source: Am J Clin Nutr. 2026 Jun 20;124(2):101401. doi: 10.1016/j.ajcnut.2026.101401 (PMC13494110; doi:10.1016/j.ajcnut.2026.101401)
Supplement: Supplementary file 1 [file mmc1.docx]

**Supplementary Materials**

**Contents**

- Participant flow chart
- Drink composition and perception
- ROI masks, ROI CBF tables and plots
- Gastric content volume
- Subjective ratings

Assessed for eligibility n=47

Excluded (not meeting criteria) n=12

Included n=35

Withdrew prematurely (n=2) Withdrew after two sessions (n=2) Withdrew after one session (n=1)

For analysis (six sessions completed) n=30

**Supplementary Figure 1**. Participant flow chart.

**Drink composition and perception**

**Supplementary Table 1.** Drink composition.

**Supplementary Figure 2**. Estimated marginal means ± SE for liking and sweetness ratings for the six treatments, adjusted for treatment order. Liking did not differ among the sweet drinks except for Monk fruit which was liked modestly less than Sucrose (difference 9.49±3.73, P_FDR_ = 0.036). All drinks except Monk fruit (difference 7.30±3.73, P_FDR_= 0.13) were liked significantly more than water (all differences > 12.7±3.7, all P_FDR_<0.003). Perceived sweetness of all drinks was greater than that of water (all differences > 44±3.5, all P_FDR_<0.001). Sweetness did not differ significantly among most flavored drinks (all P_FDR_>0.2) with the exception of Monk fruit and Allulose + stevia which were rated modestly less sweet than Sucrose (difference for both 9.4±3.4, P_FDR_ = 0.017). Different letters (a / b / c) indicate that treatments differ significantly (P_FDR_<0.05).

**ROI masks, ROI CBF tables and plots**

*ROI mask*

**Supplementary Figure 3**. Illustration of the region of interest masks of the hypothalamus, ventral striatum (Nucleus Accumbens) and dopaminergic midbrain (Ventral Tegmental Area, Substantia Nigra), overlaid on axial crossections.

*ROI CBF analysis tables*

**Supplementary Table 2.** Mean (SD) CBF values (ml/min/100 g) for the whole brain (global grey matter CBF) and for the primary ROIs.

| **ROI**^1^ | **Treatment** | **Timepoint** | | |
| --- | --- | --- | --- | --- |
|  |  | *Baseline* | *T5* | *T30* |
| **Global CBF** | Sucrose | 43.95 (7.38) | 41.84 (6.86) | 41.80 (6.97) |
|  | Sucralose | 42.79 (6.82) | 40.52 (6.46) | 40.32 (6.65) |
|  | Stevia | 42.68 (7.11) | 40.77 (6.73) | 40.03 (6.74) |
|  | Monk fruit | 42.30 (7.52) | 39.69 (7.12) | 39.51 (6.28) |
|  | Allulose + stevia | 42.64 (7.79) | 40.03 (7.19) | 41.23 (7.74) |
|  | Water | 43.14 (8.16) | 40.97 (7.67) | 40.19 (8.13) |
| **Hypothalamus**^2^ | Sucrose | 41.00 (7.20) | 40.78 (7.60) | 39.63 (7.93) |
|  | Sucralose | 39.40 (8.00) | 38.18 (6.75) | 38.28 (7.78) |
|  | Stevia | 40.20 (7.21) | 39.40 (7.46) | 38.29 (7.51) |
|  | Monk fruit | 38.64 (7.30) | 39.16 (8.38) | 37.44 (7.24) |
|  | Allulose + stevia | 40.30 (7.80) | 37.51 (7.99) | 38.65 (8.41) |
|  | Water | 39.09 (8.72) | 36.63 (8.69) | 38.38 (8.71) |
| **Nacc**^1, 2^ | Sucrose | 55.31 (9.77) | 52.69 (10.00) | 53.05 (10.15) |
|  | Sucralose | 52.46 (8.11) | 50.90 (8.35) | 51.83 (7.03) |
|  | Stevia | 51.66 (6.92) | 52.02 (7.93) | 50.42 (9.24) |
|  | Monk fruit | 51.78 (9.97) | 48.89 (9.03) | 49.44 (7.95) |
|  | Allulose + stevia | 51.91 (8.61) | 50.56 (8.47) | 51.29 (9.31) |
|  | Water | 53.03 (9.43) | 50.05 (8.32) | 51.44 (9.09) |
| **Substantia nigra**^2^ | Sucrose | 25.74 (4.29) | 24.60 (5.04) | 24.91 (5.84) |
|  | Sucralose | 24.87 (4.71) | 24.15 (5.23) | 23.87 (5.11) |
|  | Stevia | 24.50 (4.64) | 24.28 (4.12) | 23.42 (6.44) |
|  | Monk fruit | 24.59 (4.84) | 23.83 (5.74) | 23.73 (4.75) |
|  | Allulose + stevia | 25.14 (5.57) | 23.45 (4.83) | 24.46 (6.61) |
|  | Water | 25.10 (5.71) | 23.39 (5.72) | 23.77 (5.31) |
| **VTA**^1, 2^ | Sucrose | 32.00 (6.11) | 31.72 (8.37) | 29.52 (7.07) |
|  | Sucralose | 32.66 (5.73) | 30.83 (6.41) | 31.33 (6.58) |
|  | Stevia | 30.95 (5.96) | 29.22 (6.12) | 29.04 (8.45) |
|  | Monk fruit | 30.04 (6.03) | 29.44 (6.91) | 30.18 (6.78) |
|  | Allulose + stevia | 30.48 (8.04) | 28.23 (7.26) | 29.93 (8.77) |
|  | Water | 31.51 (8.02) | 30.48 (9.36) | 30.38 (7.44) |

^1^Nacc, nucleus accumbens; ROI, region of interest; VTA, ventral tegmental area

For the primary ROI CBF analysis on the hypothalamus and reward-related ROIs (ventral striatum (NAcc), midbrain (VTA and substantia nigra)) we used linear mixed models (lme) with the treatment effect of interest (delta CBF) as a fixed factor and baseline CBF, treatment order and sex as covariates. Below tables are shown for the different comparisons made.

**Supplementary Table 3**. Comparison 1) Each drink’s CBF at T=30 min compared with its own baseline (treatment effect)

| **ROI** | **Treatment** | **Estimate** | **Confidence interval** | **P_FDR_** |
| --- | --- | --- | --- | --- |
| Hypothalamus | Allulose + stevia | 0.31 | (-4.26, 4.87) | 0.8906 |
|  | Monk fruit | 1.23 | (-3.40, 5.85) | 0.8871 |
|  | Stevia | 1.92 | (-2.63, 6.47) | 0.8871 |
|  | Sucralose | 0.69 | (-3.86, 5.25) | 0.8906 |
|  | Sucrose | 1.25 | (-3.30, 5.81) | 0.8871 |
|  | Water | 2.71 | (-1.86, 7.27) | 0.8871 |
| Nacc | Allulose + stevia | 2.09 | (-1.67, 5.85) | 0.3629 |
|  | Monk fruit | 1.71 | (-2.10, 5.52) | 0.3655 |
|  | Stevia | 2.39 | (-1.44, 6.21) | 0.3629 |
|  | Sucralose | 3.07 | (-0.74, 6.88) | 0.3312 |
|  | Sucrose | 1.94 | (-1.84, 5.72) | 0.3629 |
|  | Water | 4.47 | (0.71, 8.23) | 0.1299 |
| Subst. nigra | Allulose + stevia | 0.30 | (-2.64, 3.24) | 0.9357 |
|  | Monk fruit | 1.86 | (-1.11, 4.84) | 0.8875 |
|  | Stevia | 0.12 | (-2.82, 3.05) | 0.9357 |
|  | Sucralose | 0.96 | (-1.97, 3.89) | 0.8875 |
|  | Sucrose | 1.17 | (-1.77, 4.10) | 0.8875 |
|  | Water | 0.78 | (-2.16, 3.72) | 0.8875 |
| VTA | Allulose + stevia | 0.29 | (-5.17, 5.75) | 0.9573 |
|  | Monk fruit | 4.20 | (-1.33, 9.74) | 0.2624 |
|  | Stevia | -0.14 | (-5.60, 5.32) | 0.9573 |
|  | Sucralose | 6.19 | (0.73, 11.65) | 0.1665 |
|  | Sucrose | -4.43 | (-9.89, 1.04) | 0.2624 |
|  | Water | 3.19 | (-2.28, 8.66) | 0.3643 |

**Supplementary Table 4**. Comparison 2) Each drink’s change in CBF at T=30 min compared with that of Sucrose (main treatment difference).

| **ROI** | **Contrast** | **Estimate** | **Confidence interval** | **P_FDR_** |
| --- | --- | --- | --- | --- |
| Hypothalamus | (Allulose + stevia) - Sucrose | -0.94 | (-6.79, 4.90) | 0.9923 |
|  | Monk fruit - Sucrose | -0.03 | (-5.93, 5.87) | 0.9923 |
|  | Stevia - Sucrose | 0.67 | (-5.19, 6.52) | 0.9923 |
|  | Sucralose - Sucrose | -0.56 | (-6.41, 5.28) | 0.9923 |
|  | Water - Sucrose | 1.45 | (-4.43, 7.33) | 0.9923 |
| Nacc | (Allulose + stevia) - Sucrose | 0.15 | (-4.56, 4.87) | 0.9486 |
|  | Monk fruit - Sucrose | -0.23 | (-4.98, 4.53) | 0.9486 |
|  | Stevia - Sucrose | 0.45 | (-4.34, 5.24) | 0.9486 |
|  | Sucralose - Sucrose | 1.13 | (-3.63, 5.89) | 0.9486 |
|  | Water - Sucrose | 2.53 | (-2.20, 7.26) | 0.9486 |
| Subst. nigra | (Allulose + stevia) - Sucrose | -0.86 | (-4.35, 2.62) | 0.9071 |
|  | Monk fruit - Sucrose | 0.70 | (-2.82, 4.22) | 0.9071 |
|  | Stevia - Sucrose | -1.05 | (-4.54, 2.45) | 0.9071 |
|  | Sucralose - Sucrose | -0.21 | (-3.69, 3.28) | 0.9071 |
|  | Water - Sucrose | -0.39 | (-3.89, 3.11) | 0.9071 |
| VTA | (Allulose + stevia) - Sucrose | 4.72 | (-1.73, 11.16) | 0.1877 |
|  | Monk fruit - Sucrose | 8.63 | (2.12, 15.13) | **0.0243** |
|  | Stevia - Sucrose | 4.28 | (-2.17, 10.73) | 0.1915 |
|  | Sucralose - Sucrose | 10.62 | (4.18, 17.06) | **0.0070** |
|  | Water - Sucrose | 7.61 | (1.14, 14.08) | **0.0357** |

**Supplementary Table 5**. Comparison 3) Each sweet drink’s change from baseline CBF at T=5 min compared with that of water (effect of sweetness)

| **ROI** | **Contrast** | **Estimate** | **Confidence interval** | **P_FDR_** |
| --- | --- | --- | --- | --- |
| Hypothalamus | (Allulose + stevia) - Water | 2.46 | (-3.23, 8.14) | 0.3945 |
|  | Monk fruit - Water | 6.20 | (0.57, 11.84) | 0.1185 |
|  | Stevia - Water | 4.46 | (-1.19, 10.11) | 0.2018 |
|  | Sucralose - Water | 2.95 | (-2.70, 8.61) | 0.3795 |
|  | Sucrose - Water | 5.74 | (0.07, 11.42) | 0.1185 |
| Nacc | (Allulose + stevia) - Water | 4.37 | (-0.02, 8.76) | 0.1278 |
|  | Monk fruit - Water | 0.76 | (-3.62, 5.15) | 0.7305 |
|  | Stevia - Water | 5.52 | (1.14, 9.89) | 0.0697 |
|  | Sucralose - Water | 3.33 | (-1.06, 7.72) | 0.2273 |
|  | Sucrose - Water | 2.08 | (-2.33, 6.48) | 0.4412 |
| Subst. nigra | (Allulose + stevia) - Water | 1.37 | (-1.51, 4.25) | 0.3472 |
|  | Monk fruit - Water | 2.95 | (0.08, 5.83) | 0.1093 |
|  | Stevia - Water | 3.47 | (0.60, 6.34) | 0.0901 |
|  | Sucralose - Water | 2.59 | (-0.28, 5.47) | 0.1282 |
|  | Sucrose - Water | 2.05 | (-0.83, 4.94) | 0.2017 |
| VTA | (Allulose + stevia) - Water | 1.54 | (-6.15, 9.24) | 0.8658 |
|  | Monk fruit - Water | 5.05 | (-2.63, 12.73) | 0.3261 |
|  | Stevia - Water | 0.32 | (-7.34, 7.98) | 0.9343 |
|  | Sucralose - Water | 7.59 | (-0.11, 15.29) | 0.2558 |
|  | Sucrose - Water | 6.41 | (-1.30, 14.12) | 0.2558 |

**Supplementary Table 6**. Comparison 4) Each drink’s change in CBF from T=5 to T=30 compared with that of Sucrose.

| **ROI** | **Contrast** | **Estimate** | **Confidence interval** | **P_FDR_** |
| --- | --- | --- | --- | --- |
| Hypothalamus | (Allulose + stevia) - Sucrose | 2.48 | (-4.87, 9.82) | 0.7353 |
|  | Monk fruit - Sucrose | -0.98 | (-8.39, 6.43) | 0.7943 |
|  | Stevia - Sucrose | 2.02 | (-5.34, 9.37) | 0.7353 |
|  | Sucralose - Sucrose | 2.06 | (-5.29, 9.40) | 0.7353 |
|  | Water - Sucrose | 6.94 | (-0.52, 14.39) | 0.3398 |
| Nacc | (Allulose + stevia) - Sucrose | -1.95 | (-7.94, 4.04) | 0.8683 |
|  | Monk fruit - Sucrose | 1.18 | (-4.85, 7.22) | 0.8734 |
|  | Stevia - Sucrose | -2.68 | (-8.76, 3.39) | 0.8683 |
|  | Sucralose - Sucrose | -0.23 | (-6.27, 5.80) | 0.9392 |
|  | Water - Sucrose | 5.12 | (-0.93, 11.18) | 0.4827 |
| Subst. nigra | (Allulose + stevia) - Sucrose | -0.15 | (-4.42, 4.11) | 0.9435 |
|  | Monk fruit - Sucrose | -0.41 | (-4.71, 3.89) | 0.9435 |
|  | Stevia - Sucrose | -2.48 | (-6.76, 1.79) | 0.9435 |
|  | Sucralose - Sucrose | -0.75 | (-5.01, 3.51) | 0.9435 |
|  | Water - Sucrose | 1.82 | (-2.50, 6.14) | 0.9435 |
| VTA | (Allulose + stevia) - Sucrose | 9.73 | (0.75, 18.70) | **0.0344** |
|  | Monk fruit - Sucrose | 10.13 | (1.09, 19.18) | **0.0344** |
|  | Stevia - Sucrose | 10.40 | (1.42, 19.38) | **0.0344** |
|  | Sucralose - Sucrose | 9.69 | (0.72, 18.65) | **0.0344** |
|  | Water - Sucrose | 14.45 | (5.37, 23.54) | **0.0101** |

Whole brain analysis ROI treatment by time interaction effects (Table 2)

**Supplementary Figure 4**. Peak voxel mean ± SE adjusted CBF differences based on the whole-brain analysis for the clusters in the left amygdala (A) and left putamen (B) showing a significant time by treatment interaction. For this visualisation the differences for all treatments are shown. T5/T30, time of measurement in minutes after the start of consumption. The colored bar shows the color-coding of the T-values for each voxel which are superimposed on an anatomical brain MRI (MNI152 template). * Interaction peak P_FWE_ < 0.005, small volume corrected.

**Gastric content volume**

| **Treatment:** | **Baseline** | **T = 25 min** | **T = 45 min** |
| --- | --- | --- | --- |
| **Sucrose** |  | | |
| **Sucralose** |  | | |
| **Stevia** |  | | |
| **Monk fruit** |  | | |
| **Allulose + stevia** |  | | |
| **Water** |  | | |

**Supplementary Figure 5**. Illustration of stomach MRI scan time series for all treatments (one participant). Shown is the same axial cross-section. Stomach content is highlighted in red.

**Supplementary Figure 6**. Mean ± SD gastric content volume for all treatments per timepoint.

**Supplementary Table 7**. Estimated marginal mean differences ± SE of gastric volume (ml) between treatments for each timepoint, based on the linear mixed model with baseline, sex and treatment order as covariates.

| **Contrast** | **T=25 min** | | **T=45 min** | |
| --- | --- | --- | --- | --- |
|  | **difference** | **P_FDR_** | **difference** | **P_FDR_** |
| Sucrose - Sucralose | 44.7 ± 13.0 | 0.0015 | 60.0 ± 13.0 | <0.001 |
| Sucrose - Stevia | 30.3 ± 13.0 | 0.0280 | 62.4 ± 13.0 | <0.001 |
| Sucrose - Monk fruit | 43.9 ± 12.9 | 0.0015 | 57.3 ± 12.9 | <0.001 |
| Sucrose - (Allulose + stevia) | -26.5 ± 12.9 | 0.0508 | 25.8 ± 12.8 | 0.0736 |
| Sucrose - Water | 76.1 ± 13.2 | <0.001 | 70.0 ± 13.2 | <0.001 |
| Sucralose - Stevia | -14.3 ± 13.3 | 0.3250 | 2.4 ± 13.3 | 0.8537 |
| Sucralose - Monk fruit | -0.8 ± 13.1 | 0.9511 | -2.6 ± 13.1 | 0.8537 |
| Sucralose - (Allulose + stevia) | -71.2 ± 13.1 | <0.001 | -34.2 ± 13.0 | 0.0196 |
| Sucralose - Water | 31.5 ± 13.4 | 0.0280 | 10.0 ± 13.4 | 0.6226 |
| Stevia - Monk fruit | 13.5 ± 13.2 | 0.3277 | -5.1 ± 13.2 | 0.8074 |
| Stevia - (Allulose + stevia) | -56.8 ± 13.2 | <0.001 | -36.6 ± 13.0 | 0.0133 |
| Stevia - Water | 45.8 ± 13.4 | 0.0015 | 7.5 ± 13.4 | 0.7173 |
| Monk fruit - (Allulose + stevia) | -70.4 ± 13.0 | <0.001 | -31.5 ± 12.9 | 0.0286 |
| Monk fruit - Water | 32.3 ± 13.3 | 0.0259 | 12.6 ± 13.3 | 0.5131 |
| (Allulose + stevia) - Water | 102.6 ± 13.3 | <0.001 | 44.2 ± 13.2 | 0.0028 |

**Subjective ratings**

**Supplementary Figure 7**. Mean ± SD subjective ratings over time.
